# Grief as a neuroendocrine transition: Insights from perinatal loss

**DOI:** 10.1111/jne.70262

**Published:** 2026-09-02

**Authors:** Clare Killikelly, Eva‐Maria Stelzer, Rita T. Amiel Castro

**Affiliations:** ^1^ Division of Clinical Intervention & Global Mental Health, Department of Psychology University of Zurich Zurich Switzerland; ^2^ Faculty for Psychology University of Basel Basel Switzerland

**Keywords:** grief, neuroendocrine, perinatal loss, treatment

## Abstract

Grief is an all‐encompassing human experience that reverberates across mind, brain, and body. Historically, the scientific study of grief has been rooted primarily in psychological frameworks. Pioneering work has begun to elucidate how attachment loss is encoded not only psychologically but also biologically, and this may be particularly significant during the perinatal period.

## 1 INTRODUCTION

Grief is an all‐encompassing human experience that reverberates across mind, brain, and body.^1^ It is deeply personal yet profoundly interpersonal, shaped by relationships, meanings, and the broader social worlds in which loss occurs.^2^, ^3^, ^4^, ^5^ Historically, the scientific study of grief has been rooted primarily in psychological frameworks, with a strong emphasis on cognitive processes, attachment dynamics, and social functioning.^6^, ^7^ These approaches have yielded critical insights into how individuals adapt to loss, highlighting variability in trajectories and the role of resilience and context. However, comparatively less attention has been paid to the underlying neurobiological and physiological processes that accompany bereavement. In recent years, this gap has begun to narrow, with emerging research pointing to the complex interplay between affective, neuroendocrine, and stress‐related systems in shaping grief responses. Pioneering work by researchers such as Mason and Duffy^8^ and O'Connor and Seeley^9^ has begun to elucidate how attachment loss is encoded not only psychologically but also biologically, involving dysregulation in systems such as the hypothalamic–pituitary–adrenal (HPA) axis, inflammatory responses, and neural circuits associated with reward and social pain.

Grief may be understood as a fundamental, transformative human emotion—arguably an apex experience that confronts individuals with existential questions about identity, meaning, and continuity.^10^ Like other pivotal life transitions such as childbirth, puberty, matrescence, and menopause, grief represents a moment in which the body and self‐undergo profound reorganization.^11^, ^12^ These transitions are marked not only by psychological change but also by significant neuroendocrinological shifts that shape emotional experience, cognition, and behavior.^13^, ^14^ Increasingly, scholars are beginning to conceptualize grief through this lens: not solely as a response to loss, but as a biopsychosocial transition with identifiable physiological signatures. This perspective opens new avenues for understanding both adaptive and maladaptive grief trajectories, including conditions such as prolonged grief disorder.^15^

Despite these advances, critical gaps remain. In particular, the intersection of grief with perinatal loss has received limited empirical attention. The loss of a pregnancy or infant occurs within a uniquely neuroendocrine context, yet research has rarely integrated these biological dimensions into models of grief. We argue that conceptualizing grief, especially in the context of perinatal loss, as a neuroendocrinological transition represents a promising and necessary frontier. Advancing this line of inquiry will require interdisciplinary collaboration to more fully capture the embodied, relational, and cultural complexity of grief and to inform more nuanced and effective approaches to care.

## 2 CURRENT STATE OF THE FIELD

Neuroendocrine and hormonal processes play a central role in shaping psychological responses to perinatal loss, a form of bereavement characterized by the death of a fetus or infant during pregnancy (e.g., miscarriage) or shortly after birth (e.g., stillbirth, neonatal death). Perinatal loss profoundly disrupts biological systems that normally support maternal–infant attachment, stress regulation, and mood in the perinatal period; consequently, the disruption of these processes through loss may contribute to heightened vulnerability to adverse mental health outcomes, including depression, anxiety, and prolonged grief.^16^, ^17^ Current work highlights alterations in the oxytocin (OXT) system and HPA axis as central to these outcomes. These are only two elements of a complex system that interact across many levels of genetics, physiology, psychology and context, however they provide a first understanding of the involvement of neurobiology in grief.

A key neuroendocrine system implicated in or perhaps unique to perinatal grief is the oxytocinergic system. OXT, a neuropeptide involved in lactation, bonding, maternal behavior, and stress regulation, undergoes substantial upregulation during pregnancy and the postpartum period.^18^ Experimental evidence from animal models indicates that offspring loss during this sensitive window disrupts OXT signaling pathways, particularly in brain regions associated with emotion regulation and attachment. These alterations may impair adaptive coping mechanisms and intensify grief‐related behaviors.^18^, ^19^ Human data on OXT after perinatal loss remain scarce, but reviews emphasize that disruption of OXT‐mediated bonding and anti‐stress functions is a plausible mechanism linking loss to prolonged grief and affective disorders.^15^, ^16^ At the same time, the corticotropin‐releasing factor (CRF) system—central to the regulation of the HPA axis—appears to be dysregulated following perinatal loss. Heightened CRF activity may contribute to sustained stress responses, anxiety‐like behaviors, and impaired emotional recovery.^15^

The HPA axis represents an interface between neuroendocrine function and psychological distress. Systematic reviews of grief and bereavement show elevated mean cortisol, higher morning cortisol, and flattened diurnal slopes in bereaved individuals, moderated by grief severity, depression, and relational closeness.^15^ Following perinatal loss, this system may become dysregulated, with evidence suggesting either hyperactivation or blunted cortisol responses depending on the timing and context of assessment. Such dysregulation has been associated with symptoms of depression and chronic stress, indicating that maladaptive HPA axis functioning may underlie persistent grief responses.^17^

In addition to OXT and cortisol, other hormonal systems are likely involved. Rapid declines in reproductive hormones such as estrogen and progesterone following delivery are well‐established contributors to postpartum mood disturbances.^20^ In the context of perinatal loss, these abrupt hormonal shifts occur in the absence of the expected caregiving experience, potentially exacerbating emotional dysregulation. Neuroactive steroids, including allopregnanolone, which modulate GABAergic activity and influence mood stability, may also play a role, although empirical evidence in bereaved populations remains limited.^21^

Findings from human cohort studies further underscore the psychological and physiological burden associated with perinatal loss.^22^, ^23^ Women who experience stillbirth or neonatal death report significantly elevated levels of anxiety, depression, and stress during a subsequent pregnancy compared to non‐bereaved counterparts. While these studies are primarily observational, they align with neurobiological models suggesting that disruptions in stress‐regulatory and bonding‐related systems contribute to adverse outcomes.

## 3 FUTURE RESEARCH, MODELS IN DEVELOPMENT, TREATMENT PROSPECTS

Emerging theoretical models conceptualize perinatal loss as a complex multidimensional condition involving disruption of maternal endocrine pathways, attachment systems, immune signaling, and trauma symptoms.^22^, ^24^, ^25^ Current animal and human perinatal loss models^16^, ^18^, ^22^, ^26^ propose a mismatch between maternal neurobiological pathways commonly seen during pregnancy and the postpartum period and the physical absence of the infant. Typically, maternal endocrine and neural changes facilitate attachment, lactation, and caregiving in mothers; however, in the case of perinatal loss, these pathways are partly disrupted or remain active (despite the loss), contributing to dysregulated emotion processing and stress regulation, as well as prolonged grief reactions following offspring loss.

Perinatal loss is increasingly recognized as a public health bereavement concern with long‐term psychosocial implications.^22^ However, no specific treatment recommendations exist for perinatal loss and its neurobiological underpinnings are not fully understood. The convergence of findings across animal and human models calls for translational and mechanistic frameworks that integrate endocrine, immune, and psychological pathways. As outlined above, the most promising key players include oxytocin signaling, the HPA axis and inflammatory processes. In a next step, knowledge of how maternal neurobiological changes impact adjustment and contribute to prolonged grief disorder should be utilized to develop therapeutic strategies for perinatal loss. Additionally, future research should seek to overcome current methodological inconsistencies (e.g., small sample sizes, study heterogeneity) and address personal and contextual factors (e.g., mother's sociocultural background) as well as the specific loss type (e.g., miscarriage, stillbirth, recurrent pregnancy loss) which likely produce distinct neuroendocrine profiles. Longitudinal studies that explore the timing and symptom dynamics over the course of grief and the progression to prolonged grief disorder would be essential.

Given that perinatal loss is linked to heightened psychological distress and disrupted neuroendocrine alterations, future treatment prospects should target both psychological and neurobiological processes (see Table 1). Evidence‐based psychological interventions such as cognitive behavioral therapy or mindfulness‐based interventions are not only directly linked to reduced symptoms of depression, post‐traumatic stress disorder (PTSD), or anxiety in perinatal loss^27^ but may also act indirectly by reducing chronic HPA‐axis activation and improving stress regulation, thereby normalizing disrupted neuroendocrine functioning. Similar beneficial effects on maternal HPA‐axis functioning and emotional reactivity could emerge from low‐key psychosocial interventions such as sleep restoration, physical activation, or stress‐reduction trainings. On the neurobiological side, oxytocinergic pathways are a promising therapeutic target. Oxytocin plays a central role in maternal attachment, mother–child, and social bonding in general, stress buffering, and emotional regulation.^28^ Preliminary studies suggest that oxytocin administration enhances stress regulation and may influence loss rumination and attachment processing during grief.^29^, ^30^, ^31^, ^32^ Similarly, oxytocin may improve emotional adaptation in perinatal loss. However, it might also intensify attachment‐related distress. More research is needed to disentangle the effects of oxytocin administration following offspring loss. Another therapeutic target specific to perinatal loss is the management of lactation‐related endocrine processes.^33^ Lactation suppression or personalized hormone therapy may help restore neurobiological balance in mothers.

**TABLE 1:** Hypothetical targets for perinatal loss interventions at the psychological and neurobiological levels.

Psychological processes	Neurobiological processes	Potential interventions
Grief rumination	HPA‐axis hyperactivation	CBT, MBIs, stress‐reduction training
Anxiety and hypervigilance	Elevated cortisol and autonomic arousal	CBT, mindfulness, sleep interventions
Attachment‐related distress	Oxytocinergic dysregulation	Attachment‐focused therapy, oxytocin research
Social withdrawal	Reduced social buffering	Peer support, family interventions, group‐based support
Emotional dysregulation	Neuroendocrine imbalance	Mindfulness, exercise, hormonal interventions
Distress related to lactation after loss	Persistent prolactin/lactation processes	Lactation suppression and endocrine management

Abbreviations: CBT, Cognitive behavioural therapy; MBI, mindfulness‐based interventions.

In sum, perinatal loss represents a unique intersection of biological and psychological disruption, in which neuroendocrine and hormonal systems play a pivotal role. Dysregulation in oxytocinergic and stress‐related pathways, alongside broader hormonal changes, appears to contribute to the intensity and persistence of grief, underscoring the need for biologically informed approaches to assessment and intervention.

## AUTHOR CONTRIBUTIONS

**Eva‐Maria Stelzer:** Writing – original draft; conceptualization; writing – review and editing. **Rita T. Amiel Castro:** Conceptualization; writing – original draft; writing – review and editing. **Clare Killikelly:** Conceptualization; writing – original draft; writing – review and editing.

## CONFLICT OF INTEREST STATEMENT

The authors report no conflicts of interest.

## Data Availability

Data sharing not applicable to this article as no datasets were generated or analysed during the current study.
